# Ototoxicity and otoprotection in the inner ear of guinea pigs using gentamicin and amikacin: ultrastructural and functional aspects

**DOI:** 10.1016/S1808-8694(15)30144-0

**Published:** 2015-10-18

**Authors:** Thomaz José Marra de Aquino, José Antônio Apparecido de Oliveira, Maria Rossato

**Affiliations:** a PhD at USP / FMRP, Department of Ophthalmology, Otorhinolaryngology, Head and Neck Surgery at Faculdade de Medicina de Ribeirão Preto, Universidade de São Paulo; b Associate Professor at Universidade de São Paulo, Full Professor in the Department of Ophthalmology, Otorhinolaryngology, Head and Neck Surgery at Faculdade de Medicina de Ribeirão Preto, Universidade de São Paulo; c Audiology Technician, Department of Ophthalmology, Otorhinolaryngology, Head and Neck Surgery at Faculdade de Medicina de Ribeirão Preto, Universidade de São Paulo.; d Department of Ophthalmology, Otorhinolaryngology, Head and Neck Surgery at Faculdade de Medicina de Ribeirão Preto, Universidade de São Paulo

**Keywords:** aminoglycosides/toxicity, cytoprotection, otoacoustic emissions, spontaneous, inner ear/injuries

## Abstract

Ototoxicity is still a challenge to medicine. The discovery of self-protecting endogenous mechanisms of the outer hair cells associated with their functional and ultra-structural assessment methods has opened new horizons in the understanding and controlling of these mechanisms. **Aim**: this paper aimed at establishing whether or not underdoses of gentamicin could protect the inner ear against the harmful effects of amikacin, based on these protection mechanisms and determine if the otoacoustic emission amplitudes could be associated with the level of hair cell integrity. **Materials and Methods**: Experimental study. We used 31 guinea pigs. They were injected with saline solution, gentamicin and amikacin, alone and in combinations -intramuscular injections - during 12, 30 and 42 days. The otoacoustic emissions were recorded in the beginning and at the end of the experiment, comparing it with the cochlear integrity study carried out by electron microscopy. **Results**: gentamicin underdoses did not protect the inner ear against amikacin toxicity; the reduction in otoacoustic emissions was strongly associated with an increase in hair cell lesions. **Conclusion**: these findings help understand inner ear otoprotection and ototoxicity. Establishing the correlation between the emissions amplitude an cell integrity plays an important role in the follow up of hair cell damage, with possible monitoring of ototoxicity caused by drugs in humans.

## INTRODUCTION

Ototoxicity still remains as a challenge for today”s medical science. In spite of the vast research done on the topic by renowned personnel and institutions and the increasing knowledge on the pathophysiology of ototoxicity, the inner ear lesions introduced by ototoxic agents used in human beings are yet to be satisfactorily controlled. Lesion irreversibility and associated hearing loss observed in a great deal of the patients is another critical factor from the social standpoint^1^, ^2^.

Aminoglycosides are indubitably among the most widely prescribed ototoxic drugs because of their antimicrobial effectiveness and affordability. Many studies have been conducted recently to try and find mechanisms to protect the inner ear against the harmful effects of these medications, producing promising results and illuminating the path to solving this most severe problem.

An increasingly important new line of research is looking at the endogenous inner ear protection mechanisms. Antioxidant enzymes such as catalase, superoxide dismutase, glutathione peroxidase, glutathione reductase, and glutathione S-transferase have been targeted. Glutathione, a tripeptide found in all mammal cells, has also been found to have protective action over in vitro outer hair cells against cytotoxicity^3^, ^4^.

It was recently discovered that small non-toxic doses of amikacin or gentamicin, administered for a certain period of time as habituation previously to the administration of ototoxic amounts of the same aminoglycoside used during habituation, protect the cochlea against the dose-dependent harmful effects of the medication, possibly due to the triggering of endogenous self-defense mechanisms^5^, ^6^. In such studies, scanning electron microscopy and otoacoustic emissions are among the most broadly assessment methods given their practicality and ease of operation.

This paper aims to contribute to the understanding of the pathophysiological inner ear protection mechanisms against drug-induced ototoxicity. It specifically looks into habituation underdoses of gentamicin and whether these can offer any protection against ototoxicity by amikacin, and tries to determine if distortion product otoacoustic emission response amplitude can be used to assess the level of morphologic integrity of outer hair cells in guinea pigs.

## MATERIALS AND METHOD

Thirty-one male albino guinea pigs were used in this study. Two of the 62 cochleae were discarded due to technical difficulties experienced during preparation, and 60 were thus included in the study. Albino guinea pigs were elected because they are easy to handle, cochlear dissection and manipulation is not challenging, and experimental drugs and anesthesia can be injected intraperitoneal, intramuscular, or subcutaneously.

Animals were handled according to The Guide for the Care and Use of Laboratory Animals issued by the Institute of Laboratory Animal Resources, Commission on Life Sciences, National Research Council^7^. TRhe experimental protocol also followed the ethical principles of the Brazilian College on Animal Experimentation (Colégio Brasileiro de Experimentação Animal - COBEA) and was approved by the Ethics Committee for Animal Experimentation at the Ribeirão Preto Medical School under permit 086/2004.

### Inclusion Criteria

The animals were selected from the Central Biotherium of the Ribeirão Preto Medical School at the University of São Paulo. They had Preyer reflex and weighed between 400 and 600 grams. After a 24-hour auditory rest, the guinea pigs were assessed and submitted to external otoscopy. Animals with signs of external otitis or acute otitis media, earwax that could not be removed, inflammatory processes in the external ear meatus, or ear canals too narrow to fit the OAE measurement probe, were excluded. Guinea pigs with earwax that could be removed were kept. All included animals were then submitted to hearing screening through distortion product otoacoustic emission (DPOAE) tests carried out in a soundproof booth under anesthesia with ketamine (65 mg/Kg). Animals with present DPOAE in both ears were selected.

### Study Groups

The guinea pigs were divided into four groups:

GROUP 1 CONTROL - SALINE SOLUTION: six guinea pigs - twelve cochleae; 0.9% saline solution intramuscular injections; one single dose per day of same volume as the animal weight based dose of amikacin for 30 consecutive days.
GROUP 2 CONTROL - GENTAMICIN: five guinea pigs - ten cochleae; intramuscular injections of gentamicin; single daily dose of 10mg/kg/day for 30 consecutive days.
GROUP 3 CASE - AMIKACIN: ten guinea pigs - eighteen cochleae; intramuscular injections of amikacin; single daily dose of 400mg/kg/day for 12 consecutive days.
GROUP 4 CASE - GENTAMICIN + AMIKACIN: ten guinea pigs - twenty cochleae; intramuscular injections of gentamicin, single daily dose of 10mg/kg/day from day 1 to 30, followed by intramuscular injections of amikacin, single daily dose of 400mg/kg/day, for another 12 days (day 31 to 42).

The elected gentamicin dosages were considered not to introduce significant inner ear damage, as observed by Maudonnet6 and confirmed by our control group 2. The purpose was to promote outer hair cell habituation previously to exposure to a potentially ototoxic drug - amikacin.

Amikacin was administered in a potentially ototoxic dosage as seen in Oliveira et al.^5^ and Canedo^8^ and confirmed by the results gathered from case group 3. Amikacin was used to test for possible improvements on outer hair cell (OHC) resistance against damage secondary to habituation with gentamicin. Case group 4 was treated with this purpose.

### Hearing Functional Assessment

Hearing functional assessment was performed through distortion product otoacoustic emission tests done using equipment ILO 92 CAE System Otodynamics LTD. Guinea pigs were sedated with ketamine before the tests. Before DPOAE acquisition the animals underwent external ear meatus and tympanic membrane examination. Only guinea pigs with present DPOAE were included in the study.

Control group 1 (0.9% saline solution), control group 2 (gentamicin) and case group 3 (amikacin) subjects had their DPOAE tests done on the first day of the study and then one day after drug or saline solution injection. Group 4 (gentamicin + amikacin) subjects were tested for DPOAE on day 1 when they were administered gentamicin, on day 31 when gentamicin was stopped and amikacin initiated, and on day 43, 24 hours after the last amikacin injection and immediately before slaughter. In this study we used sound intensities of 70 dB SPL. Triggering stimulus may range from 0 to 70 dB SPL and can be measured within the range of 500 to 8000Hz. The method described herein is the same adopted by Hyppolito et al.^9^, ^10^.

The higher pitch otoacoustic emissions were deemed as more relevant to quantitatively assess the functional status of the outer hair cells located in the base of the cochlear canal, where aminoglycoside-induced ototoxicity lesions are more prevalent. DPOAE was considered as present or absent. OAE amplitude variation was also analyzed between the beginning and end of the experiment and compared against inner ear morphological analysis findings of each guinea pig.

### Morphologic Assessment

**Figure 1:**
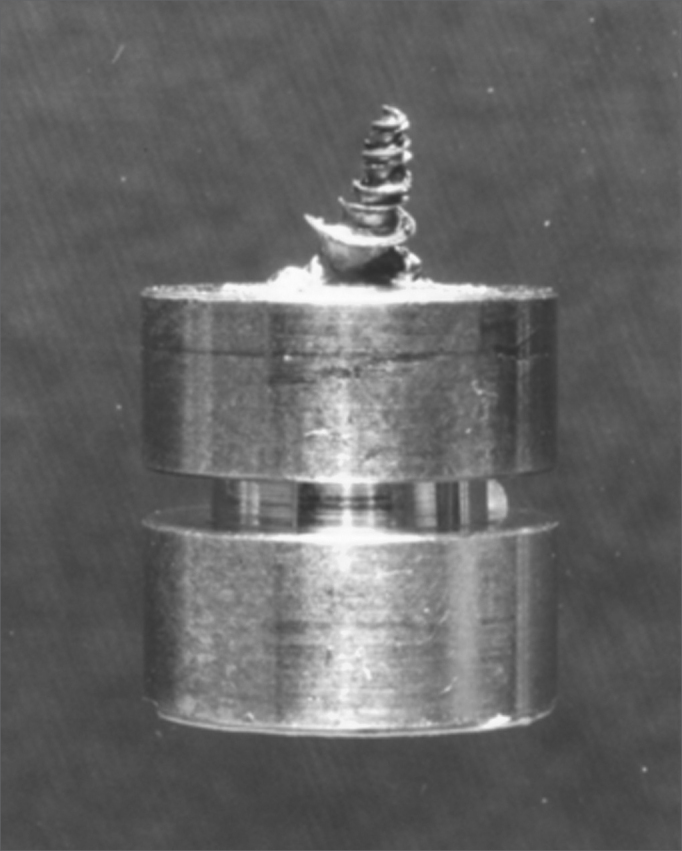
Specimen support showing a cochlea prepared for study in the scanning electron microscope.

On the day following the last injection given to all four groups and after DPOAE tests, the guinea pigs were given lethal intraperitoneal injections of thiopental sodium (Thionembutal®) and their ethmoid bullae were bilaterally opened to expose their cochleae. After the tympanic bullae were opened, the cochleae were prepared for analysis under scanning electron microscopy. The main steps were: fixation using 2.5% glutaraldehyde, 0.1M phosphate buffer solution, and osmium tetroxide; dehydration with ethanol; specimens were dried using a Bal-Tec CPD 030 device through the critical point method, structure metallization by vaporization of a thin layer of gold using a Bal-Tec SCD 050 device, and fixation of the cochleae in a metallic specimen support (Fig. 1).

**Figure 2:**
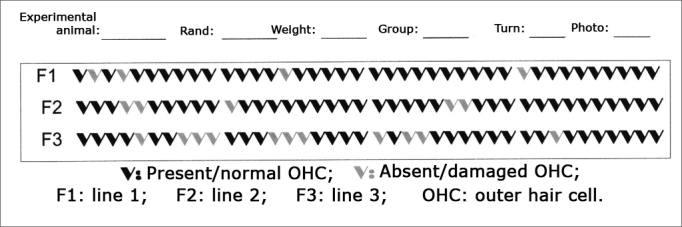
Cochleogram showing the field of study of outer hair cells under SEM examination.

After the previously mentioned processes were completed, the specimens were taken to a scanning electron microscope (SEM). The microscope used was a JEOL SCANNING MICROSCOPE - JSM 5200. Structural analysis was standardized and a field in the middle third of the cochleae was studied using 500x magnification. As the surface of the organ of Corti was scanned the image was frozen, photographed, and studied through a cochleogram (Fig. 2). Hair cell integrity was defined based on the analysis of their stereocilia. Hair cells with perfect stereocilia were considered healthy. Hair cells with missing or deformed stereocilia were considered damaged^5^, ^9^, ^10^. Normal and damaged outer hair cells were then counted to allow for statistical comparisons between groups. The percentage of normal and damaged hair cells in the first three turns of each cochlea was recorded for each group and later compared against distortion product otoacoustic emission test results.

DPOAEs were considered as either present or absent. Statistical analysis was also done to determine the possible correlations between DPOAE response amplitude reduction and OHC damage degree. Response amplitude was measured using the average DPOAE intensity at 2kHz and 5kHz established in the initial and final tests, compared and categorized into four types (all guinea pigs had present OAE in the initial test). The response types were categorized as follows:

Response A OAE present in the final test with amplitude (average response intensity) varying by as much as 25% in relation to the initial test.
Response B OAE present in the final test with reduced response amplitude varying between 25%+ and 50% in relation to the initial test.
Response C OAE present in the final test with reduced response amplitude varying over 50% in relation to the initial test.
Response D OAE not found in the final test.

The four response types were compared against morphologic findings (normal and damaged OHC under SEM) and their correlation was statistically analyzed.

### Statistical Analysis

Software programs SPSS - Statistical Package for Social Sciences - Release 10.0 and R Program Statistical Computing - Release 2.2.1 were used to statistically treat the data.

For statistical and comparison purposes, damaged and normal outer hair cell counts were performed in the middle third of the first three cochlear turns from images magnified 500 times under scanning electron microscopy.

## RESULTS

**Figure 3:**
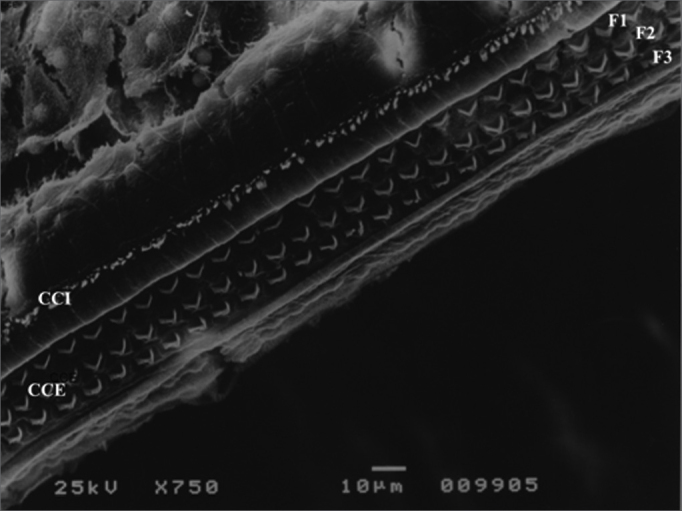
Microscope image from normal organ of Corti from a guinea pig in group 1. CCI: inner hair cell; CCE: outer hair cell; F1: row 1; F2: row 2; F3: row 3. 750x magnification.

**Figure 4:**
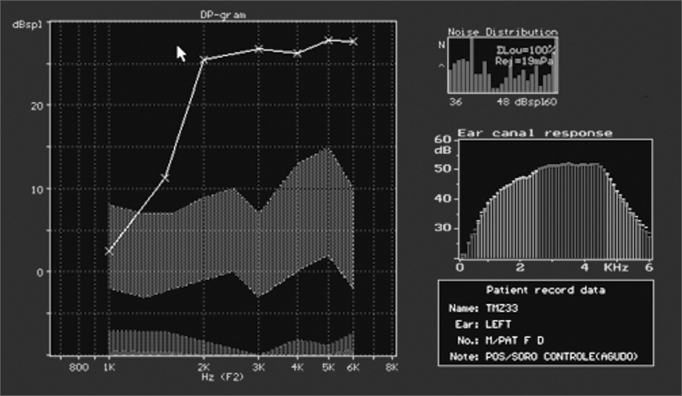
Normal DPOAE (distortion product otoacoustic emission) test results of guinea pig in group 1.

Group 1 (0.9% saline solution for 30 days) and group 2 (gentamicin 10 mg/kg/day for 30 days) did not present significant outer hair cell damage under SEM examination in any of their cochleae. In group 1, 100% of the outer hair cells were normal at the end of the experiment, against 94.2% in group 2. Functional assessment showed DPOAE was present at the end of 30 days in both ears of all guinea pigs included in the two groups (Fig. 3 and 4).

**Figure 5:**
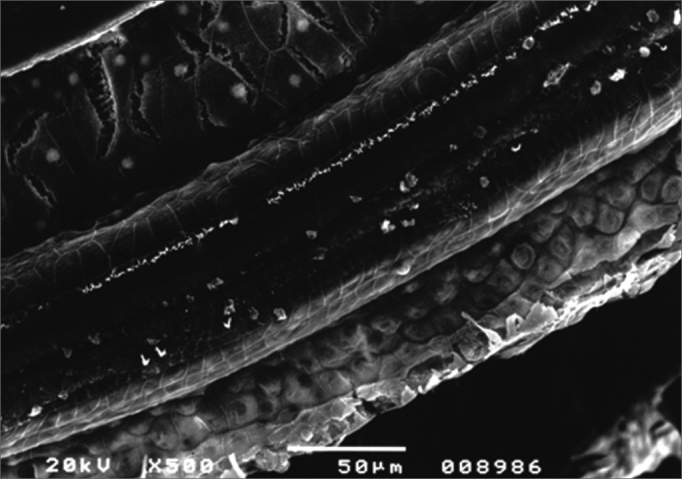
Microscope image from organ of Corti of guinea pig from group 3 showing extensive damage in all three rows with almost no outer hair cells left. 500x magnification.

Guinea pigs in group 3 (amikacin for 12 days) and group 4 (gentamicin for 30 days + amikacin for 12 days) had damaged outer hair cells in all cochleae in different degrees (Fig. 5).

OHC damage in group 3 ranged between 4.3% and 96.9% (mean damage 46.4%) for the 18 cochleae included in the group (53.5% of normal cells). Damage occurred predominantly in the first two turns of the cochlea, i.e., they had fewer normal outer hair cells than the third turn. Damage was more intense on the first row of outer hair cells, followed by the second row.

Damage ranged between 5.7% and 100% in group 4, with mean OHC damage of 53.2% (46.8% of normal OHC) for the 20 cochleae of the group. Damage occurred predominantly in the two first turns, as they had lower counts of normal outer hair cells. Damage was also more intense on the first row of outer hair cells, followed by the second row (Table 1).

**Table 1:** Mean normal outer hair cells per group.

GROUP 1	GROUP 2	GROUP 3	GROUP 4
100%	94,2%	53,5%	46,8%

A total of 20,100 outer hair cells from 60 cochleae and 4 groups were analyzed, averaging 335 outer hair cells per cochlea.

**Figure 7:**
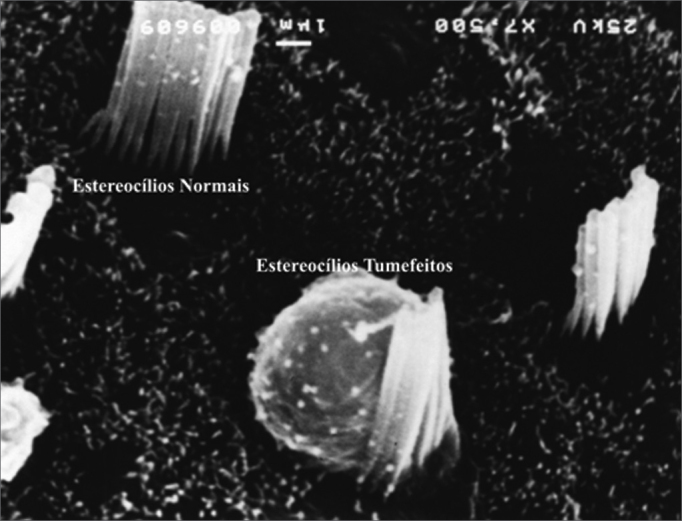
Microscope image from organ of Corti of guinea pig showing normal stereocilia next to a cell with damaged tumefied stereocilia. 7,500x magnification.

**Figure 8:**
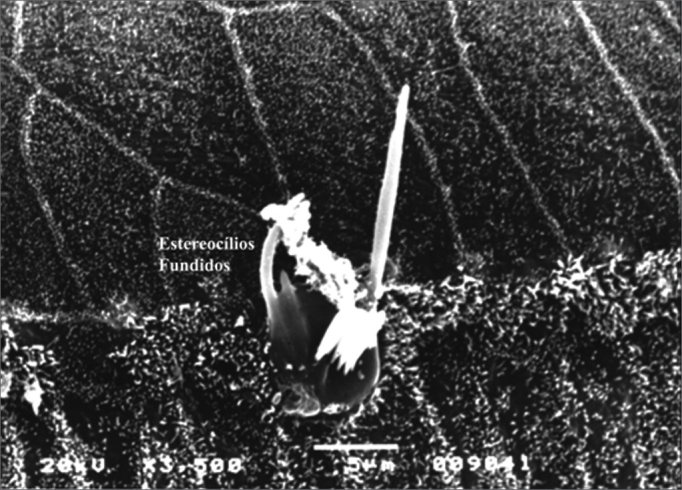
Microscope image from organ of Corti of guinea pig showing fused stereocilia. 3,500x magnification.

In terms of hair cell architecture, the main types alteration in the damaged outer hair cells were absence of hair (the most frequent finding), distorted pattern from ‘v” to ‘w”, hair cell tumefaction and fusion (Fig. 7 and 8).

**Figure 6:**
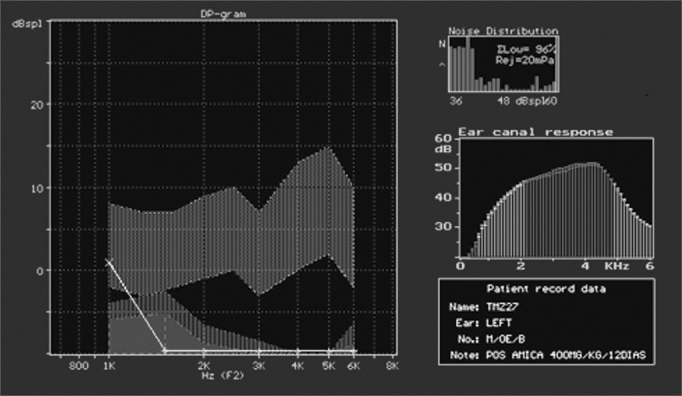
DPOAE (distortion product otoacoustic emission) test results of guinea pig in group 3, showing absence of response after drug administration.

In groups 1 (saline solution) and 2 (gentamicin), as reported, there was no significant damage and DPOAEs were present and normal. In group 3 (n=18; acute damage by amikacin) DPOAEs were present in 11 ears. These eleven ears had a mean value for normal OHC of 75.5%. DPOAEs were absent in seven ears at the end of the experiment, and their mean value for preserved OHC was 21.6% (Fig. 6). In group 4 (n=20; underdoses of gentamicin followed by amikacin) DPOAEs were present in 12 ears, with a mean value of normal OHC of 68%. DPOAEs were absent in eight ears at the end of the experiment, and their mean value for preserved OHC was 18.9%. The association between normal and damaged OHC and presence or absence of DPOAE Is shown in Table 2.

**Table 2:** Mean normal outer hair cell (OHC) level in ears with present and absent otoacoustic emissions (OAE) at the end of the experiment for groups 3 and 4.

	Mean preserved OHC - Group 3.	Mean preserved OHC - Group 4
Ears with OAE	75,5%	68,0%
Ears without OAE	21,6%	18,9%

Damage severity decreased in significance from the first and second turns to the apex and from the first row of outer hair cells to the third. There was no statistically significant difference in damage between the two first turns of the cochlea. Although inner hair cells were not included in the study, their observation indicated little damage in all groups.

The apical turn was not considered as it processes lower sounds, and ototoxic drugs involve mainly higher frequencies. This turn also presents naturally disarrayed hair cells, which hinders thorough anatomic assessment. Inner hair cells were not considered in this study as they are less susceptible to ototoxicity and difficult to count individually when compared to outer hair cells.

One of the objectives of this study was to compare group 3 (ototoxic dose of amikacin) to group 4 (ototoxic dose of amikacin preceded by habituation doses of gentamicin) findings to tell whether gentamicin would protect group 4 subjects from amikacin ototoxicity.

Groups 3 and 4 were tested for normality using the Shapiro-Wilk test. Group 3 was found not to follow a normal distribution. Therefore the non-parametric Mann-Whitney U test was used.

The comparison between normal and damaged OHC in groups 3 and 4 had p=0.501, showing that there was no statistically significant difference between groups (Table 3). Gentamicin did not protect guinea pig outer hair cells against amikacin ototoxicity. Statistical analysis was also done on the findings from the first cochlear turn of subjects on these two groups, and tests were repeated for turns 2 and 3. Likewise, no statistically significant difference was found between groups (p=0.65 for first cochlear turns; p=0.83 for second turns; p=0.36 for third turns). A statistical comparative test was then run between DPOAE presence and absence for groups 3 and 4. For statistical calculation purposes, all OAEs - even the ones with reduced amplitude when compared to the initial examination - were considered as present. As this is a categorical variable, the chi-square test was used. Results are as follows: p=0.793 - thus no statistically significant difference between groups 3 and 4 in relation to ear functional assessment by DPOAE, matching morphological analysis.

**Table 3:** Results of statistical analysis on the number of normal OHC at the end of the experiment for groups 3 and 4 *.

	MEAN	STANDARD DEVIATION	MEDIAN	P
GROUP 3	54,60	33,59	59,80	
GROUP 4	48,35	37,82	47,20	

				0,50

* Mann-Whitney”s U test. Statistical Package for Social Sciences.

In order to verify the existence of statistically significant correlations between DPOAE response amplitude/intensity reduction and increase in OHC damage, we calculated Spearman”s non-parametric ratio, whose result (r = 0.81) revealed a high and significant correlation (p<0.001) between OAE amplitude/intensity reduction and number of normal outer hair cells in guinea pig inner ears (Table 4).

**Table 4:** Spearman ratio between OAE intensity variation and degree of OHC damage.

			VAR 0001	VAR 0002
Spearman”s Rho	VAR 0001	Correlation Ratio	1,000	0,812
		Sig (Bi caudal)	0,0	0,000
		N	59	59
	VAR 0002	Correlation Ratio	0,812	1000
		Sig (Bi caudal)	0,00	

		N	59	59

Morphologic values and OAE response categories were compared through the Kruskal-Wallis test (non-parametric ANOVA) which showed X2 = 39.75 (p<0.001), revealing therefore a significant difference between morphologic values associated with the various response types (Tables 5 and 6).

**Table 5:** Kruskal-Wallis test for morphologic values between OAE response categories.

	OAE response	N	*Mean Rank*
Normal OHC	A	32	41,58
	B	4	35,88
	C	8	18,25
	D	15	10,00

	Total	59	

Statistical Package for Social Sciences - Release 10.0

**Table 6:** Kruskal-Wallisa, b test - statistical analysis between response types.

	Normal OHC
Chi-Square	39.74
Df	3
Assymp. Sig.	0,000

Statistical Package for Social Sciences - Release 10.0

Kruskal Wallis test;

Variable Group: OAE response

Dunn”s post hoc test was applied in order to check which differences between response types were significant. We found that Responses A and B have similar value and that both are significantly higher than Responses C and D, and that Response C has significantly higher value than Response D: (A=B)>C>D. This shows a correlation between DPOAE amplitude reduction and damage increase or reduced normal OHC count (Table 7).

**Table 7:** Dunn”s Post Hoc test. Significance analysis between DPOAE response and normal OHC. VAR normal OPHC.

VAR normal OPHC				
VAR OAE RESP	N	1	alpha = 0,05 2	3
D	15	20,20		
C	18		45,86	
B	4			87,12
A	32			93,43

var: variable; resp: response; Statistical Package for Social Sciences - Release 10.0

## DISCUSSION

Although reliable data on drug-induced hearing loss in the world”s population is not available, we know that it amounts to a significant occurrence, mainly in cases where aminoglycosides and chemotherapy drugs are used in a continuous fashion. Additionally, drug-induced hypoacusis is irreversible and introduces severe social and psychological burdens in the lives of patients. Therefore, more research on ototoxicity and inner ear protection is required.

Aminoglycosides are the most studied ototoxic drug, whether it is for historical reasons or clinical relevance^11^, ^12^. The countless reports on aminoglycoside ototoxicity have produced significant knowledge on inner ear anatomy, physiology, and biochemistry, thus opening the path to the development of less toxic drugs and more effective means of protecting and preventing damage against the organ of Corti.

According to Oliveira and Bernal^11^, the damage introduced by aminoglycosides in the organ of Corti affect mostly the outer hair cells and progress towards the base and then the apex of the cochlea. In the basal turns, the first row of OHC is the first to be damaged, followed by the second and third rows. The sequence of damage coincides with the height of outer hair cells, being the basal turn the first to be damaged, followed by the second, third, and then the apical cells1. The stria vascularis may also be involved, and marginal cells may be structurally affected^11^.

The identification of sensorial cell and organ of Corti endogenous defense mechanisms in the form of antioxidant and detoxification enzymes catalase, superoxide-dismutase, glutathione-peroxidase, reductase, S-transferase, and glutathione, gave a fresh breath to the research done on ototoxicity and inner ear protection, notably on protection against oxygen-reactive substances^13^, ^14^.

It has been recently shown that habituation dosages (lower, non-toxic dosages) of aminoglycosides administered before exposure to ototoxic levels of the same drug used for habituation protects the cochlea against ototoxic damage. This effect has been confirmed for amikacin^5^, ^8^ and gentamicin^6^. The inferred explanation is that lower, non-toxic dosages of a potentially ototoxic agent would stimulate the cochlea to produce endogenous defense substances, thus preparing it to better handle higher dosages and thus mitigate damage. Scan electron microscopy was used in these studies to examine the organ of Corti after drug injection, combined with otoacoustic emission (OAE) functional analysis.

Our study attempted, for the first time, to analyze the effects of using one aminoglycoside in non-toxic levels in a habituation regimen to prevent ototoxic damage caused by another aminoglycoside. We used the same approach adopted in previous publications in the literature, however using two different drugs instead of one same medication. The drugs used in our study were gentamicin - a known vestibulotoxic aminoglycoside - during habituation and amikacin - a predominantly cochleotoxic drug - as the agent to produce acute damage. Functional assessment was carried out through distortion product otoacoustic emission tests done at the end of treatment, following the method described in other publications6,9. In the morphological analysis the damage observed in the outer hair cells was identified as a function of absent of deformed stereocilia. Specimens were looked at in a SEM, photographed, transferred to a cochleogram, counted, and statistically treated.

Differently from the previously cited publications, we were unable to find statistically significant differences between the group using only amikacin (acute damage) and the one using gentamicin during habituation and then amikacin, both in looking at their cochleae in general and cochlear turns 1, 2, and 3 comparatively. Mean OHC damage was 46.5% (56.5% normal) for group 3 (treated only with amikacin) and 53.2% (46.8% normal) for group 4 (underdoses of gentamicin and then amikacin), thus failing to reveal a statistically significant difference (p=0.501). Therefore, no protection was observed against the ototoxic effects of amikacin when administering gentamicin in a habituation regimen. Individualized comparative analysis of cochlear turns of both groups also failed to show statistically significant differences (p=0.656 between first turns; p=0.833 between second turns; p=0.364 between third turns). The results diverged possibly due to the combination between the chosen drugs. Gentamicin was picked as the habituation drug. As it is predominantly vestibulotoxic, it was probably not able to stimulate the inner ear to produce endogenous defense substances in sufficient amounts to provide for protection against the highly harmful dosages of amikacin, a cochleotoxic drug that insults the organ of Corti. We looked at a total of 60 cochleae and 20,100 OHC (mean 335 OHC per cochlea). In our study, differently from the cited publications, we did not observe more damage in the basal cochlear turn when compared to the second turn of subjects in groups 3 and 4. This is possibly due to the high degrees of damage found in these groups, as this pattern is lost with damage progression and cochlear turns with 100% damaged OHC in all three rows are found. In some cases even the inner hair cells are involved, although less intensely.

Morphological analysis showed that the most frequent OHC damage type was absence of stereocilia, followed by stereocilia deformity (fusion and tumefaction). This finding is consistent with other publications in the literature^5^, ^6^, ^9^.

DPOAEs have been used as a method to assess hearing function in guinea pigs pro some time^9^, ^15^, ^16^, ^17^, ^18^. This test is easy to perform, affordable, and highly reproducible. Additionally, it has offered strong correlations to the morphological analysis of the organ of Corti.

Inner ear functional analysis through distortion product otoacoustic emission (DPOAE) testing showed strong correlation with SEM morphological findings. Groups 1 (saline solution) and 2 (gentamicin) did not present significant morphological damage and had present DPOAEs in all subjects. When DPOAEs were present in group 3 (n=18; acute damage by amikacin) subjects, mean normal OHC levels reached 75.5%; when they were absent, the mean normal OHC level dropped to 21.6%. When DPOAEs were present in group 4 (n=20; underdoses of gentamicin and then amikacin) subjects, the mean normal OHC level reached 68%. When they were absent, the mean normal OHC level dropped to 18.9%.

Part of the group of guinea pigs with present DPOAEs at the end of the experiment had reduced OAE response amplitude on the test done at the end of the study, when compared to the test done at the beginning (Figure 9A, Figure 9B). The guinea pigs with reduced OAE amplitude also had increased degrees of OHC damage when compared to the ones without amplitude decrease. They were categorized into four types of responses based on amplitude variation or OAE intensity and were compared for OHC integrity. A strong, significant correlation was then observed between OAE reduced amplitude/intensity and normal OHC count. This strong correlation is relevant as it shows that not only the presence, but also the variation of amplitude must be observed. We believe this is a most relevant clinical fact when considering the applications of ototoxicity monitoring in human beings.

**Figure 9A:**
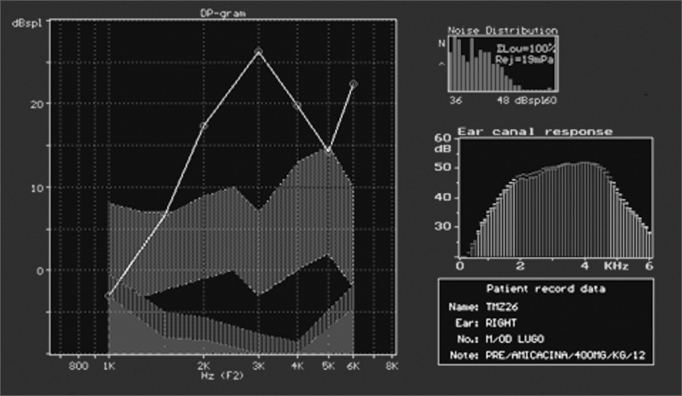
Guinea pig pre-treatment normal DPOAE test results

**Figure 9B:**
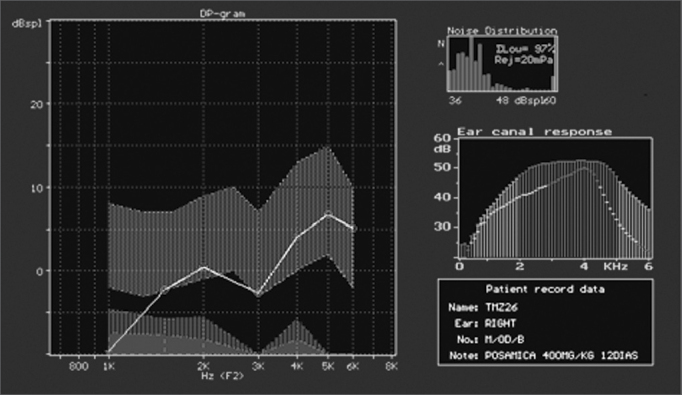
Post-treatment DPOAE test results of the same guinea pig shown in Figure 9A showing reduced response amplitude when compared to the test done previously.

The coincidence observed between normal DPOAEs and good morphological integrity, and amplitude reduction or absence of OAEs and mild to severe outer hair cell damage speak of the strength of our findings and support Chambers^19^, in that ‘the degree of permanent dysfunction is correlated to the number of destroyed or altered sensorial hair cells”.

We believe that studies on the endogenous defense mechanisms adopted by the hair cells of the organ of Corti done concurrently with genetic research^20^, ^21^, ^22^ and functional assessments form the rational path towards achieving concrete results that will enable us to better manage damage and prevent inner ear toxicity.

## CONCLUSION

Gentamicin administered in non-toxic dosages in habituation mode does not add to the protection of guinea pig inner ear outer hair cells against amikacin ototoxicity. The correlation between distortion product otoacoustic emission amplitude reduction and increased counts of damaged outer hair cells in guinea pigs is statistically significant.
